# Self-Reported Burden in Elderly Patients With Localized Prostate Cancer Treated With Stereotactic Body Radiation Therapy (SBRT)

**DOI:** 10.3389/fonc.2019.01528

**Published:** 2020-01-22

**Authors:** Nima Aghdam, Abigail Pepin, Michael Carrasquilla, Colin Johnson, Malika Danner, Marilyn Ayoob, Thomas Yung, Siyuan Lei, Brian T. Collins, Deepak Kumar, Simeng Suy, John Lynch, Sean P. Collins

**Affiliations:** ^1^Department of Radiation Medicine, Georgetown University Hospital, Washington, DC, United States; ^2^George Washington School of Medicine and Health Sciences, Washington, DC, United States; ^3^Department of Surgery, University of Southampton, Southampton, United Kingdom; ^4^Biotechnology Research Institute, North Carolina Central University, Durham, NC, United States; ^5^Department of Urology, Georgetown University Hospital, Washington, DC, United States

**Keywords:** SBRT, prostate cancer, burden of disease, patient reported outcome (PRO), quality of life, cyberknife

## Abstract

**Purpose:** Retaining quality of life in patients treated with SBRT for prostate cancer remains paramount. As such, balancing the benefits of treatment against the effects of therapy on elderly patients is essential. The EORTC QLQ-ELD14 (ELD-14) is a validated questionnaire with a domain dedicated to burden of illness and treatment in the elderly. The Expanded Prostate Cancer Index Composite (EPIC)-26 is a validated questionnaire which measures urinary, bowel, sexual, and hormonal symptoms. This study reports trends in self-reported burden in patients with prostate cancer treated with SBRT and reveals convergence of self-reported burden with treatment related side effects obtained from the EPIC-26 questionnaire.

**Methods:** All patients ≥70 years old, with localized prostate cancer treated with SBRT ± ADT at Medstar Georgetown University Hospital from 2013 to 2018 and had completed the ELD-14 were eligible for inclusion in this cross-sectional cohort study. Percentage of responses to questions related to disease and treatment burden were counted for each category (“not at all” and “a little” vs. “quite a bit” and “very much”). Additional demographic features were derived from available medical records. A total of 111 patients (median age of 74) responded to the ELD-14 questionnaire at onset of treatment and at the 2-year mark. Responses to EPIC questionnaires at matched follow-ups were scored and correlated with the self-reported burden domain of the ELD-14 using the Spearman correlation coefficient.

**Results:** Number of patients reporting “quite a bit” or “very much” burden from prostate cancer was 6.3% prior to treatment. This was highest at 1-month (10.8%) and decreased to 9.0% at 24 months post-SBRT (*X*^2^ = 3.836, *p* = 0.6986). By comparison, 3.6 and 5.4% reported “quite a bit” or “very much” burden from treatment at start of treatment and 24 months, respectively (*X*^2^ = 1.046, *p* = 0.9838). Patient reported treatment burden was found to converge well with individual domains of EPIC-26. Patients undergoing ADT experienced more burden than their non-ADT counterparts.

**Conclusions:** This cross-sectional study suggests a minority of patients reported high burden from their clinically localized prostate cancer or from their SBRT treatment. Self-reported burden converged well with lower EPIC scores in multiple domains.

## Introduction

Critical advances in the precise delivery of high doses of radiation to the prostate have made ultrahypofractionation a promising option for patients with localized prostate cancer. While clinical trials are underway, early findings from multi-institutional consortium have shown excellent outcomes (1–3). At the same time, careful assessment of patient reported quality of life measures (QoL) has provided important guidance in management of early and late treatment related toxicities. Given that prostate cancer is often a curable disease with many efficacious modalities available to patients, it is imperative to consider burden of treatment in deciding between the various options.

Patient burden is multifactorial related to the anxiety of diagnosis, logistics of treatment, and associated treatment related side effects. Validated instruments to assess patient's subjective burden of disease and treatment are not commonly used in prostate cancer clinics. The EORTC QLQ-ELD14 (ELD-14) questionnaire was developed to supplement the QLQ-C30 and address health-related quality of life in elderly patients with cancer. The questionnaire was previously validated in individuals over 70 with cancer, and assesses mobility, family support, anxiety, burden of disease, and illness (4). We were interested in assessing how these domains impact our population of elderly prostate cancer patients undergoing radiation therapy. As such, we introduced the EORTC-QLQ-ELD14 (ELD-14) questionnaire in our clinic. The Expanded Prostate Cancer Index Composite (EPIC)-26 is a validated instrument that evaluates urinary, bowel, sexual function and bother, and hormonal outcomes (5). We have employed this questionnaire in our clinic and have previously reported on the use of EPIC-26 to assess for patient reported outcomes (5–8). Critical gaps remain in our understanding of patients' perceived burden of disease and treatment and its correlation with urinary, bowel, sexual, and hormonal symptoms (6). In this report, we seek to correlate the ELD-14 burden domains with the EPIC-26 domains on patient reported outcomes. To our knowledge, this is the first report on subjective disease and treatment burden for patients with localized prostate cancer who have undergone SBRT.

## Patient Selection

The Georgetown University Institutional Review Board (IRB) approved this single institution prospective quality of life (QoL) study (IRB#: 2009-510), and it meets the requirements for protection of human rights. For this cross-sectional cohort study, all individuals diagnosed with localized prostate cancer who received SBRT at MedStar Georgetown University Hospital from 2007 to 2018 were eligible for inclusion. Patients were stratified using the D'Amico risk group classification (9). Patients who received androgen deprivation therapy were included in the cohort. Twenty-four months of follow-up were required to be included in the analysis. Patients were required to have completed the QLQ-ELD-14 and EPIC-26 prior to treatment and during the majority of subsequent follow-ups for 24 months. Only individuals ≥70 years old were eligible for inclusion in the study given the validation of the ELD-14 questionnaire was done in this patient population.

## SBRT Treatment Planning and Delivery

Simulation, contouring, and treatment planning were conducted based on a previously described institutional protocol (10). One week after placement of 4 to 6 gold fiducial markers in the prostate, patients underwent a CT simulation of the pelvis. The bladder, prostatic urethra, membranous urethra, and rectum were contoured (SC). Inverse planning was generated with prescription dose of 35–36.25 Gy in five fractions using 6-MV photons calculating on MultiPlan version software (Accuray Inc., Sunnyvale, USA). Dose volume histograms were constructed to meet clinically established dose objectives and constraints for OARs. Specifically, treatment was delivered using the CyberKnife robotic radiosurgical system (Accuray Inc., Sunnyvale, CA, USA). Fiducial tracking using continuous orthogonal x-rays was employed to account for intrafractional target motion.

## Follow-Up and Statistical Analysis

Cross-sectional assessment of patients' quality of life was conducted using QLQ- ELD14 and EPIC-26 on the first day of treatment and at 1, 3, 6, 9, 12, 18, and 24 mons following treatment during subsequent routine follow-ups. Our analysis focuses on single item questions of burden of illness and burden of treatment in the QLQ-ELD14. All ELD14 responses were converted into a burden score between 0 and 35 by averaging the responses at a given time point and using a calculation provided in the supplemental materials. In this questionnaire, higher scores represent increased burden. These scores were stratified by individuals who underwent ADT at the start of treatment and who did not. We identified the percentage of responses to each of the burden domain questions, including those who responded “not at all,” “a little,” “quite a bit,” or “very much” at each time point. Finally, the score for each EPIC-26 item was converted into a standardized value in accordance with standard EPIC-26 scoring instructions (11). We separated cohorts into individuals who reported no burden and patients who reported any burden. Based on these cohorts, averages of the standardized values were calculated in each the domain of interest at each time point (11). Lower domain scores represented increased burden. Questions 1-5, 6a-7, 8a-12, and 13a-13e of the EPIC26 short form represented the urinary, bowel, sexual, and hormonal domains, respectively. We sought to assess convergence validity between the ELD14 burden domain score and EPIC-26 domains using the Spearman Correlation (4). In assessing for the convergence validity, we anticipated that the scales on the ELD14 burden domain and EPIC-26 domains would be correlated. Spearman Correlation was considered significant if the absolute value of the coefficient was >0.35. The statistics were performed in SPSS (Version 23.0. Armonk, NY: IBM Corp). Chi-square tests were performed and figures were made on Prism v8.3 (GraphPad Software, San Diego, CA).

## Results

**Figure 1:**
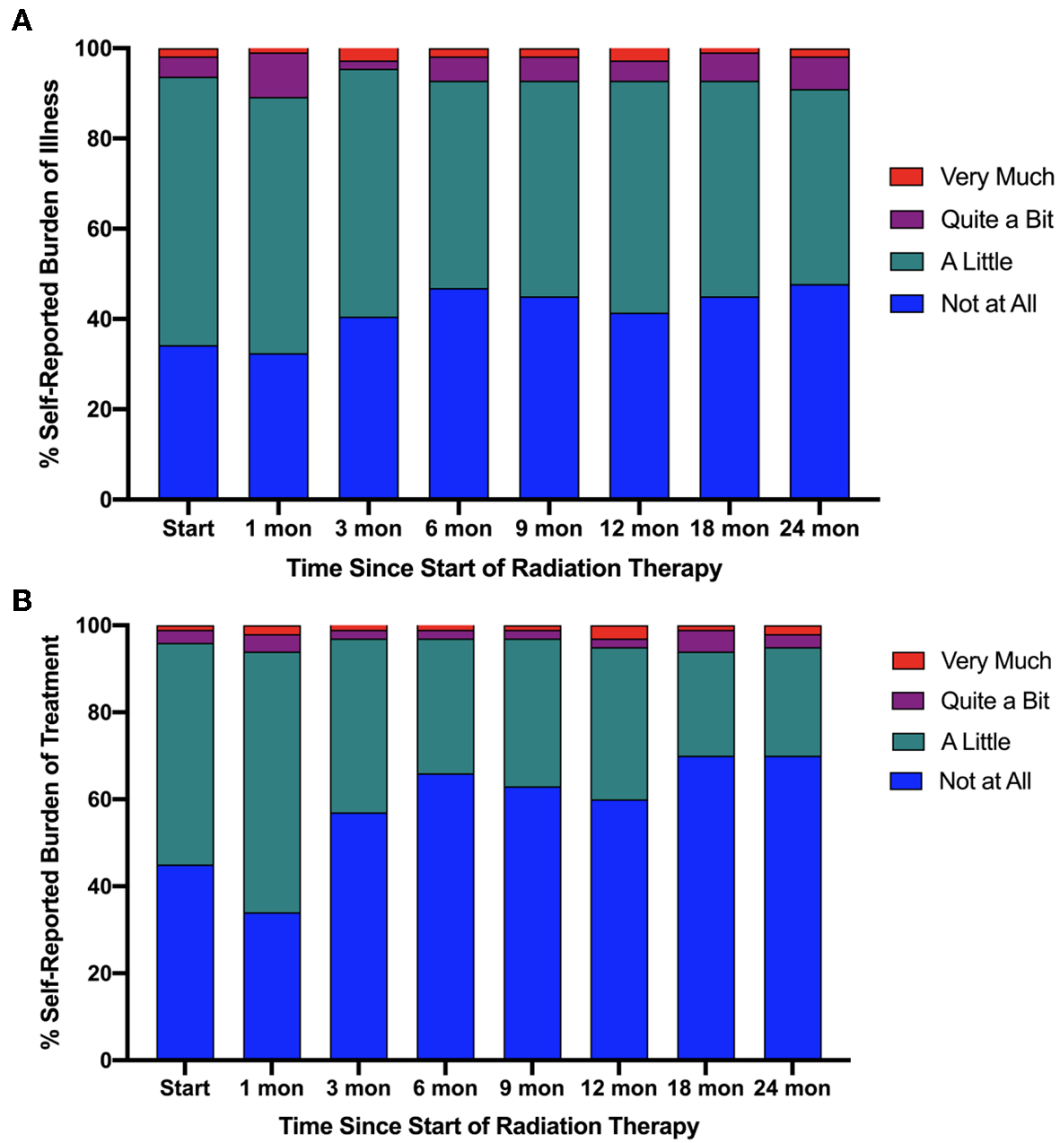
Self-reported **(A)** burden of illness and **(B)** burden of treatment based on the ELD-14 questionnaire stratified as percentage response by very much burden (red), quite a bit of burden (purple), a little burden (green), or no burden at all (blue) against time from start of radiation therapy.

**Figure 2:**
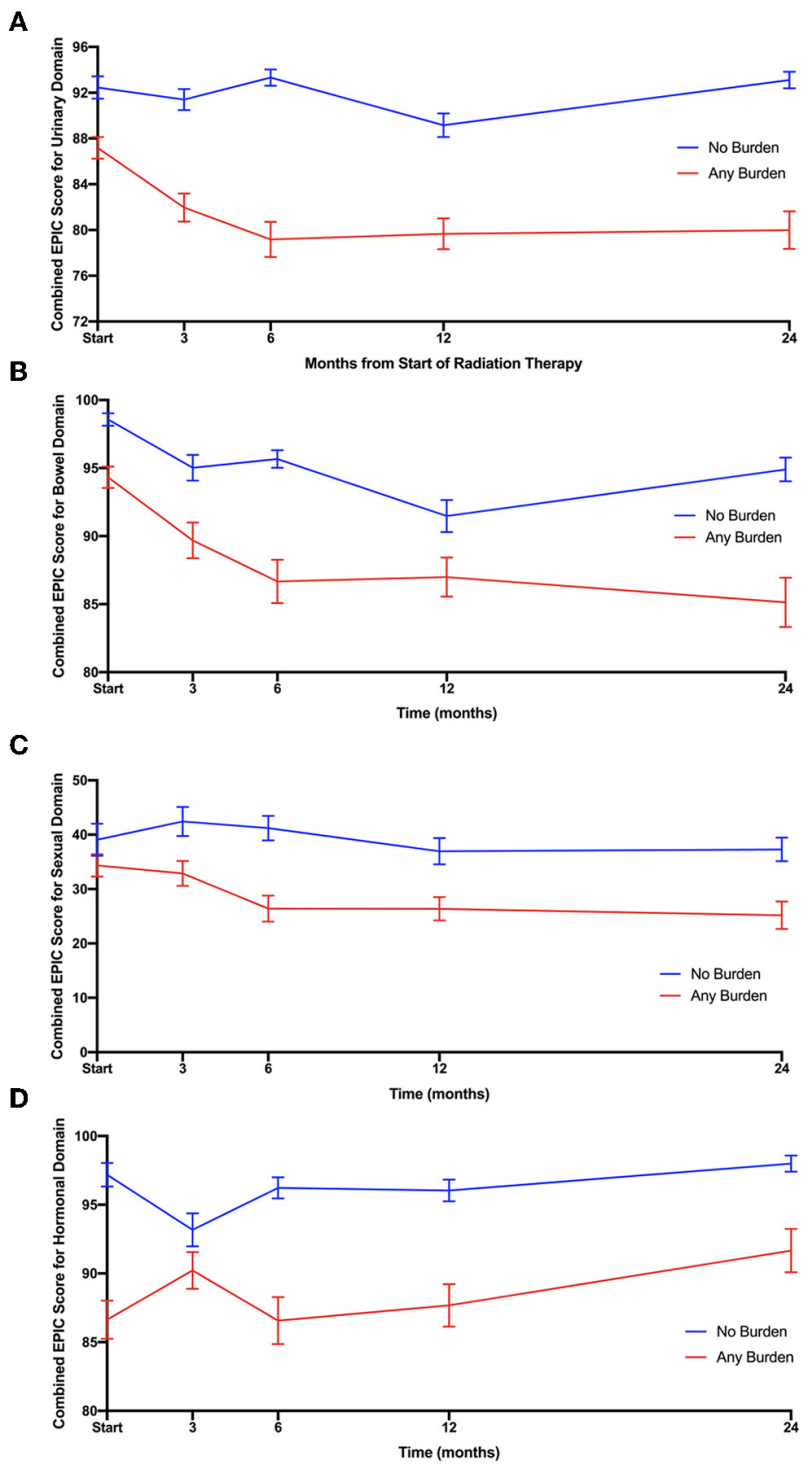
Responses to **(A)** urinary, **(B)** bowel, **(C)** sexual, and **(D)** hormonal domains of EPIC 26 over time since beginning radiation therapy. The responses are stratified by patients reporting any burden (red) and patients reporting no burden (blue) in ELD14 questionnaire.

Between August 2013 and August 2018, 111 patients that completed both ELD-14 and EPIC-26 at the onset of treatment and at 24 months post-treatment were eligible for inclusion in this study. Patient characteristics are presented in Table 1. Proportional responses to the burden of treatment and disease questions in ELD-14 are shown in Figure 1. Prior to treatment, 6.3% of patients reported “quite a bit” or “very much” burden from prostate cancer. This number increased to 10.8% at 1-month post-SBRT and reached its nadir at 3 months (4.5%). By 24 months, 9.0% reported “quite a bit” or “very much” burden from their disease. There was no significant difference of burden of disease over time (*X*^2^ = 3.836, *p* = 0.6986). Likewise, prior to treatment, 3.6% of patients reported “quite a bit or “very much” burden from treatment for their prostate cancer. This burden increased to 6.3% at 1 month and reached its nadir at 3 months post-SBRT (3.6%). At 24 months, 5.4% reported “quite a bit” or “very much” burden from treatment (Figure 1). There was no significant difference of burden of treatment across time (*X*^2^ = 1.046, *p* = 0.9838). In patients who report any burden on the ELD-14 questionnaire, the EPIC-26 sexual, urinary, bowel, and hormonal domain scores are worse than those who report no burden at all time points (Figure 2). The EPIC-26 scores in the urinary domain begin to decline in the months following treatment in patients experiencing burden (Figure 2A). By 6 months post-SBRT, the mean value is 79.2 in patients. This remains stable at 24 mon at 80. In the bowel domain, symptoms in the patients reporting burden are most at 6 and 18 months following treatment and improve at 9 months (Figure 2B). In the sexual domain, symptoms are poor at the start of treatment and remain stable in the months following treatment (Figure 2C). Finally, in the hormonal domain, patients experiencing any burden experience the most hormonal symptoms 6 months post-SBRT, but the symptoms improve relative to baseline (86.6) by 24 months at 91.7 (Figure 2D).

**Table 1:** Patient Characteristics.

	**Patient percentage (*n* = 111)**
Age (years)	Median 74 (70–87)
Race	
White	68.4 (77)
Black	22.5 (25)
Other	8.1 (9)
Pretreatment PSA (ng/ml)	Median 10.03 (1.6–100)
T stage	
T1c–T2a	73.9 (82)
T2b–T2c	26.1 (29)
Gleason score	
6	23.4 (26)
7	57.7 (64)
8–9	18.9 (21)
Risk group	
Low	12.6 (14)
Intermediate	64.9 (72)
High	22.5 (25)
Hormone Status	
Yes	27.0 (30)
No	73.0 (81)
SBRT dose	
35	20.7 (23)
36.25	79.3 (88)

We assessed convergence validity between the EPIC-26 domain scores and the ELD-14 burden scores using the Spearman Correlation (4). The results are reported in Table 2 and reveal significant correlation between bowel, urinary, and hormonal function at various timepoints when the coefficient >0.35. EPIC-26 urinary domain appears to have strongest correlation with the ELD-14 burden domain over nearly every time point in follow up (1, 3, 6, 9, 18, and 24 mon). In the bowel domain, there is convergence at 6 and 18 months. The sexual domain appears to have the weakest correlation with the ELD-14 burden domain. In the hormonal domain, there is convergence at 1, 6, 9, and 24 months.

**Table 2:** Convergence of ELD14 burden domain's composite score with EPIC-26 urinary, bowel, sexual, and hormonal function.

	**EPIC domain convergence with burden domain**			
	**Urinary**	**Bowel**	**Sexual**	**Hormonal**
SBRT Start	−0.16535	−0.2399	0.025495	−0.3477
1 Month	−0.5174^**^	−0.26541	−0.10523	−0.37512^**^
3 Month	−0.40595^**^	**–**0.33245	**–**0.13898	**–**0.10912
6 Month	−0.47292^**^	−0.44464^**^	**–**0.11121	−0.4568^**^
9 Month	−0.50781^**^	**–**0.18895	**–**0.19427	−0.44757^**^
12 Month	**–**0.2969	**–**0.11908	**–**0.15222	**–**0.33159
18 Month	−0.4014^**^	−0.44067^**^	**–**0.16248	**–**0.31982
24 Month	−0.48738^**^	**–**0.25163	**–**0.18681	−0.38083^**^

Bold and ^**^indicates significance of the Spearman Correlation at that time point. EPIC-26 urinary domain appears to have strongest correlation with the ELD-14 burden domain over nearly every time point in follow up. Hormonal domains revealed a resilient correlation with patient reported burden while sexual domain appears to have the weakest correlation with the ELD-14 burden domain.

**Figure 3:**
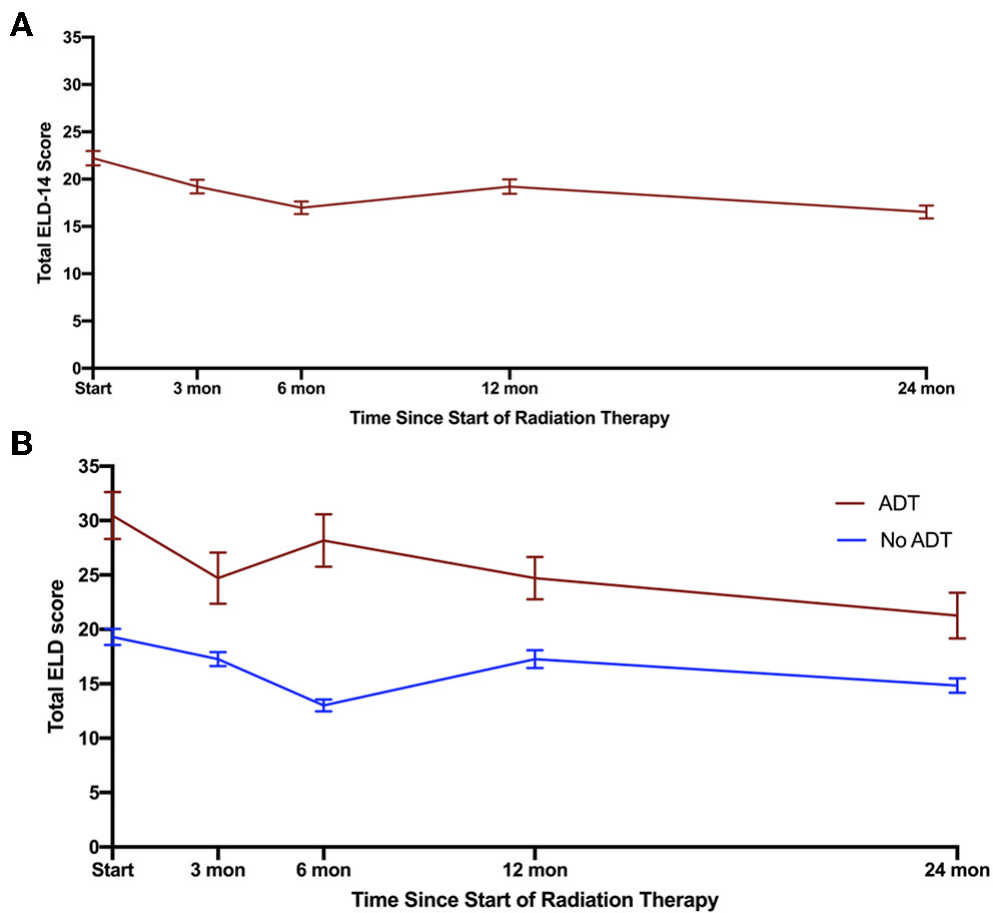
Total burden scores (including burden of disease and treatment) based on ELD for **(A)** all patients, **(B)** stratification of patients who underwent ADT (red) or did not undergo ADT (blue). Error bars represent SEM.

Given the convergence of the ELD-14 score with the EPIC domains, we stratified patients based on their ADT use (Figure 3). The total ELD-14 burden scores for all patients and burden scores stratified by ADT use are graphically demonstrated in Figure 3. The ELD-14 burden domain was significantly higher for patients who also received ADT over time. The most pronounced difference was at 6 months where the ADT cohort experiences more burden while no ADT cohort experiences the least amount of burden. Both cohorts represent less burden over time. The baseline ELD-14 scores for the ADT and the no-ADT cohorts are 30.5 and 19.3, respectively. By 24 months, the ELD-14 scores are 21.3 and 14.8, respectively.

## Discussion

In the current series, a minority of patients report high burden from disease or from treatment after undergoing SBRT for their localized prostate cancer. At its peak, 10.8% of patients report “quite a bit” to “very much” burden of illness in contrast with 6.3% reporting burden from treatment suggesting that patients attribute most of their perceived burden to their illness and not to their treatment. Interestingly, the fluctuation in the burden domain of ELD-14 mimic an expected pattern of symptomatology observed in patients treated with SBRT. Immediately after treatment, patients report high level of burden likely reflecting the higher incidence of acute toxicity in the urinary and bowel domain. The convergence of EPIC-26 and ELD-14 burden domains suggest that, symptomatic declines in bowel, sexual and urinary domains likely contribute to the incidence of self-reported burden. However, nearly 20% of patients who report “not at all” or “a little” burden of illness and treatment have similar EPIC-26 scores in bowel, urinary and sexual domain as those in the high-burden cohort. Therefore, patient's perception of burden is likely nuanced with additional elements such as sociodemographic or logistical factors.

Finally, patients who received ADT experienced greater burden over time. This is consistent with previous studies suggesting the diminished quality of life in patients who have been treated with ADT (12–14). What is surprising is that the self-reported burden in ADT cohort extended beyond the time frame in which testosterone recovery occurs (15, 16). This suggests that the deleterious side effects of ADT continue to incur a burden on patients treated for localized prostate cancer. This may include: continued fatigue, physical deconditioning as well as muscle mass loss.

There are several identifiable limitations to our study. Although data was prospectively collected at the time of treatment and follow-up, we have retrospectively chosen to parse out the “burden” domain and correlate it to the above variables. EORTC-QLQ-ELD14 is a unique instrument, which addresses several domains that are not routinely assessed in clinic; other domains including anxiety and mobility are reported elsewhere.

Furthermore, it is impossible for two questions to provide sufficient granularity in determining the nature of treatment burden in our patients. Nevertheless, as a screening mechanism, these questions appear to be highly correlated with lower score in sexual, urinary, and bowel domains of EPIC-26. As part of a questionnaire that addresses: anxiety, family support, musculoskeletal symptoms, burden of disease, and illness, this short instrument is easily deployable in a busy clinic and may offer an initial screening tool for further investigations.

## Conclusion

Our findings suggest that only a small fraction of patients experience significant burden as a direct result of their treatment as quantified by the EPIC-26. Furthermore, as expected, patients who undergo ADT appear to experience more treatment and disease related burden. However, there is a subset of patients who experience burden of treatment in domains currently not assessed with our available instruments. This is evident in the number of patients who report a significant decline in their QOL domain but without a correlating increase in their subjective measure of burden. To this end, we have initiated a pilot study in the clinic to conduct open-ended interviews with our patients who report moderate to severe burden in ELD-14. The goal of this follow-up study is to allow our patients to report what constitutes as burden of illness and treatment on their terms without an instrument to bias their interpretation of these questions. Our hope is that in doing so, we can construct a more holistic assessment of our patient's experience as they undergo treatment for their localized prostate cancer.

## Data Availability Statement

The datasets generated for this study will not be made publicly available because: Patient consent and privacy related concerns.

## Ethics Statement

The studies involving human participants were reviewed and approved by The Medstar Georgetown University Hospital Institutional Review Board (IRB) approved this single-institution prospective quality of life study (2009-510). The patients/participants provided their written informed consent to participate in this study.

## Author Contributions

NA and AP were the lead authors, who participated in data collection, data analysis, manuscript drafting, table/figure creation, and manuscript revision. TY and MA aided in clinical data collection. MD contributed to the study design and clinical data collection. SL developed the majority of patients' SBRT treatment plans and contributed to the data analysis and interpretation. SS is a senior author who organized the data and participated in its analysis. CJ contributed design of the study as well as manuscript review. MC wrote portions of the manuscript and contributed to table/figure creation. DK participated in data analysis and manuscript review. BC and JL are senior authors who aided in drafting the manuscript. SC was the principal investigator who initially developed the concept of the study and the design, aided in data collection, and drafted and revised the manuscript. All authors contributed to manuscript revision, read, and approved the submitted version.

### Conflict of Interest

SC and BC serve as clinical consultants to Accuray Inc. The Department of Radiation Medicine at Georgetown University Hospital receives a grant from Accuray to support a research coordinator. The remaining authors declare that the research was conducted in the absence of any commercial or financial relationships that could be construed as a potential conflict of interest.

## References

[1] Kishan AU Dang A Katz AJ Mantz CA Collins SP Aghdam N. Long-term outcomes of stereotactic body radiotherapy for low-risk and intermediate-risk prostate cancer. JAMA Network Open. (2019) 2:e188006. 10.1001/jamanetworkopen.2018.8006 30735235 PMC6484596

[2] Lukka HR Pugh SL Bruner DW Bahary JP Lawton CAF Efstathiou JA. Patient reported outcomes in NRG oncology RTOG 0938, evaluating two ultrahypofractionated regimens for prostate cancer. Int J Rad Oncol Biol Phys. (2018) 102:287–95. 10.1016/j.ijrobp.2018.06.008 29913254 PMC6248906

[3] Boyer MJ Rushing C Peterson B Papagikos MA Kiteley RA Lee WR Toxicity and quality of life report of a phase 2 study of stereotactic body radiation therapy (SBRT) for low- and intermediate-risk prostate cancer. Int J Rad Oncol. Biol. Phys. (2016) 96:E256–7. 10.1016/j.ijrobp.2016.06.1266

[4] Wheelwright S Darlington A-S Fitzsimmons D Fayers P Arraras JI Bonnetain F. International validation of the EORTC QLQ-ELD14 questionnaire for assessment of health-related quality of life elderly patients with cancer. Br J Cancer. (2013) 109:852–8. 10.1038/bjc.2013.407 23868003 PMC3749575

[5] Bhattasali O Chen LN Woo J Park JW Kim JS Moures R. Patient reported outcomes following stereotactic body radiation therapy for clinically localized prostate cancer. Radiat Oncol. (2014) 9:52. 10.1186/1748-717X-9-52 24512837 PMC3931491

[6] Woo Jennifer Ai-Lian Chen Leonard N Hongkun Wang Cyr Robyn A Onita Bhattasali Kim Joy S Rudy Moures. Stereotactic body radiation therapy for prostate cancer: what is the appropriate patient-reported outcome for clinical trial design? Front Oncol. (2015) 5:77. 10.3389/fonc.2015.00077. 25874188 PMC4379875

[7] Woo JA Chen LN Wang H Cyr RA Bhattasali O Kim JS. Clinical characteristics and management of late urinary symptom flare following stereotactic body radiation therapy for prostate cancer. Front Oncol. (2014) 4:122. 10.3389/fonc.2014.00122 24904833 PMC4033266

[8] Danner M Hung M Yung T Ayoob M Lei S Collins BT. Utilization of patient-reported outcomes to guide symptom management during stereotactic body radiation therapy for clinically localized prostate cancer. Front Oncol. (2017) 7:227. 10.3389/fonc.2017.00227 29085804 PMC5650639

[9] D'Amico AV Whittington R Malkowicz SB Schultz D Blank K Broderick GA Biochemical outcome after radical prostatectomy, external beam radiation therapy, or interstitial radiation therapy for clinically localized prostate cancer. JAMA. (1998) 280:969–74. 10.1001/jama.280.11.969 9749478

[10] Repka MC Guleria S Cyr RA Yung TM Koneru H Chen LN. Acute urinary morbidity following stereotactic body radiation therapy for prostate cancer with prophylactic alpha-adrenergic antagonist and urethral dose reduction. Front Oncol. (2016) 6:122. 10.3389/fonc.2016.00122 27242962 PMC4870496

[11] Sanda M Wei J Mark Litwin Scoring Instructions for the Expanded Prostate Cancer Index Composite (EPIC). (2002). Accessed online at: https://medicine.umich.edu/sites/default/files/content/downloads/Scoring%20Instructions%20for%20the%20EPIC%2026.pdf (accessed June 12, 2019).

[12] Adam S Koch-Gallenkamp L Bertram H Eberle A Holleczek B Pritzkuleit R. Health-related quality of life in long-term survivors with localised prostate cancer by therapy-results from a population-based study. Euro J Cancer Care. (2019) 28:e13076. 10.1111/ecc.13076 31050091

[13] Sanda MG Dunn RL Michalski J Sandler HM Northouse L Hembroff L. Quality of life and satisfaction with outcome among prostate-cancer survivors. N Engl J Med. (2008) 358:1250–61. 10.1056/NEJMoa074311 18354103

[14] Nguyen PL Alibhai SMH Basaria S D'Amico AV Kantoff PW Keating NL. Adverse effects of androgen deprivation therapy and strategies to mitigate them. Eur Urol. (2015) 67:825–36. 10.1016/j.eururo.2014.07.010 25097095

[15] Spiegel DY Hong JC Oyekunle T Waters L Lee WR Salama JK. A normogram for testosterone recovery after combined androgen deprivation and radiation therapy for prostate cancer. Int J Raiat Oncol Biol Phys. (2019) 103:834–42. 10.1016/j.ijrobp.2018.11.007 30419308

[16] Yuan Y Aghdam N King CR Fuller DB Weng J Chu FI. Testosterone levels and sexual quality of life after stereotactic body radiation therapy for prostate cancer: a multi-institutional analysis of prospective trials. Int J Radiat Oncol Biol Phys. (2019) 105:149–54. 10.1016/j.ijrobp.2019.05.014 31108142

